# Diphasic Sheeting Device with Cyanex-301 for Dislodging Feature of Divalent Cadmium from Industrial Effluent

**DOI:** 10.3390/ijerph192013281

**Published:** 2022-10-14

**Authors:** Liang Pei, Chunhui Wang

**Affiliations:** 1 National Engineering Technology Research Center for Desert-Oasis Ecological Construction, Xinjiang Institute of Ecology and Geography, Chinese Academy of Sciences, Urumqi 830011, China; 2 Xinjiang Key Laboratory of Environmental Pollution and Bioremediation, Xinjiang Institute of Ecology and Geography, Chinese Academy of Sciences, Urumqi 830011, China; 3 Institute of Geographic Sciences and Natural Resources Research, Chinese Academy of Sciences, Beijing 100101, China; 4 University of Chinese Academy of Sciences, Beijing 100049, China

**Keywords:** diphasic sheeting device, Cyanex-301, complemental feeding stage, complemental disintegrating stage, cadmium

## Abstract

A novel diphasic sheeting device (DSD) including complemental feeding stage and complemental disintegrating stage for dislodging features of Cd(II), was investigated. The complemental feeding stage included feeding liquor and Bis(2,4,4 trimethylamyl) dithiophosphonic acid (Cyanex-301) as the carrier in petroleum, and the complemental disintegrating stage included Cyanex-301 as the carrier in petroleum and hydrochloric acid as the disintegrating reagent. The impacts of volumetric ratio of sheeting liquor and feeding liquor(S/F), initial molarity of Cd(II) and ion intensity of the feeding liquor, pH, volumetric ratio of sheeting liquor and disintegrating reagent (S/D), molarity of hydrochloric acid liquor, Cyanex-301 molarity in the complemental disintegrating stage on dislodging of Cd(II), the virtues of DSD compared to the traditional sheeting device, the constancy of system, the reuse of sheeting liquor, and the retention of the sheeting stage were also investigated. Experimental results illustrated that the optimum dislodging conditions of Cd(II) were achieved as hydrochloric acid molarity was 4.00 mol/L, Cyanex-301 molarity was 0.150 mol/L, and S/D was 1:1 in the complemental disintegrating stage, S/F was 1:10, and pH was 5.00 in the complemental feeding stage. The ion intensity of the complemental feeding stage had no distinct impact on the dislodging feature of Cd(II). When initial Cd(II) molarity was 3.20 × 10^−4^ mol/L, the Cd(II) dislodging percentage was up to 92.9% in 210 min. The dynamic formula was inferred on the basis of the theorem of mass transferring and the interfacial chemistry.

## 1. Introduction

The dislodging of toxic metallic ions such as platinum, cadmium, cobalt, copper, chromium, gold, silver, mercury, and zinc from the natural environment has received considerable attention in recent years on account of their toxicity and carcinogenicity, which can lead to various damage to the human body. They also can be readily adsorbed by marine animals and directly enter the human food chain, thus presenting a high health risk to consumers [1]. Cadmium ions are non-biodegradable toxic heavy metals that may cause dermatitis and allergies [2,3]. According to the standards of the World Health Organization, the cadmium content in drinking water should be less than 0.01 mg/L, but the cadmium concentration in electroplating wastewater in various countries is far greater than this value, so it is expensive to treat it below the standard [4]. The major sources of cadmium contamination in water come from industrial procedures in aerospace, electro-plating, petro-chemical, battery manufacturing, mining, metallic materials, and many other industries [5]. It is necessary to remove and recover these highly toxic and non-biodegradable heavy metals in order to meet increasingly stringent environmental quality standards and promote the recycling and reuse of heavy metallic resources [6]. The conventional methods for these purposes include adsorption, sorbents (ion-exchange typically), filtration, chemical precipitation, dissolvant dislodging procedures, fluid sheeting, biological systems, reverse osmosis, evaporation, etc. [7]. Among these methods, the sedimentation method is usually not practical because the sludge produced by it must be landfilled, the relevant metals cannot be recovered, the chemical cost is high, and it is a semi-continuous process [8]. Biological systems are usually unstable and dynamically slow. The adsorbent can have high selectivity, capacity, and adsorption rate, but it can only work in semi-continuous operation, in which the adsorbent must be regenerated regularly [9,10,11]. Reverse osmosis, evaporation, and electrodialysis have no selectivity, while precipitation, ion exchange, and dissolvent dislodging allow metallic ion recovery, but are little used due to greater capital and running costs compared to the value of the recovered materials [12]. Therefore, exploring more effective and cost-effective dislodging and extraction methods to solve these troubles is necessary.

Many industries have paid close attention to fluid sheeting skills on account of their specific features in recent years [12]. The potential superiority of fluid sheeting techniques over traditional dislodging techniques and solid sheeting techniques are low capital and running costs, low energy and extractant agent consumption, high molarity factors, and high percentages [9,10,11,12,13]. Fluid sheetings can implement simultaneous dislodging and disintegrating procedures, and it has superiority of disequilibrium mass transferring and climbing-hill impact, where the solute can move from low-to-high molarity liquor. The main types of fluid sheeting systems include emulsion fluid and sheeting devices (SD). However, fluid sheeting techniques have not been used for extensive application in industry procedure [14]. One defect of emulsion fluid sheets is that the emulsion swells upon prolonged contact with the feed stream. This swelling leads to a decrease in disintegrant molarity in the water droplets, which decreases disintegration efficiency [15]. The second defect is the sheet fracture, resulting in the leakage of the contents of the water droplets into the feed stream and accompanied by a decrease in dislocation efficiency [16]. Sheeting devices have not had extensive application in industry. The most important reason for this is the constancy or durability of sheeting, on account of the loss of carrier or sheeting dissolvant from the sheeting stage that has an impact on the selectivity of the sheeting and dislodging percentage of elements [12,17,18,19,20,21].

Extraction technology and sheeting technology are better methods to treat industrial heavy metal wastewater. Especially, sheeting technologies have broad application prospects. Some scholars have used extraction methods to dislodge heavy metals and compounds, but efficiency has been very low and the effective use times have been lower [22,23,24,25]. Recently, some scholars have used new liquid sheeting technology to dislodge heavy metals [26]. However, due to the lack of supplementary solution, the dislodging efficiency has been low, the sheeting has been used fewer times, and the cost has been high [27,28]. Some people have also used the hollow fiber liquid sheeting device to study the dislodging of metal ions [29]. It was found that the dislodging percentage could be increased by 10% after the supplementary liquid was used, and the use times of the sheeting increased significantly [30]. Some people also used ion-exchange membrane to dislodge heavy metal compounds, and the effect was significantly lower than that of sheeting technology [31]. Many studies have also shown that the parameter selection of liquid sheeting technology is similar to that of extraction technology, but the dislodging efficiency is much higher [32]. Because the organic sheeting solution is volatile, the stability of the liquid sheeting and the extraction method is also affected [33]. Therefore, whether liquid sheeting technology can operate in a closed manner will be the focus of the research. The volatilization of the organic sheeting fluid is controlled, which can improve the dislodging percentage and stability [34].

Some novel fluid sheeting configurations have been investigated in order to overcome these difficulties, such as emulsion fluid sheeting and hollow fiber fluid sheeting [13,35,36] etc. In our previous work, one-stage complemental implanted fluid sheeting named dispersion implanted fluid sheeting was investigated for the dislodging of rare earth and the dislodging results were very distinct [13,14]. In this study, a new fluid sheeting technique, named the diphasic sheeting device (DSD), was explored for the dislodging and recovery of objective substances and materials from a feeding liquor. This is a new fluid sheeting procedure that has shown more virtues than traditional sheeting devices in our early work. The impacts of different experiment parameters on Cd(II) dislodging of effluent were investigated. After experimental research, this technology might be applied in practice. The selected optimal operation conditions could break through the bottleneck of water treatment and membrane technology. It could also provide scientific and theoretical support for the treatment of industrial waste residue, especially the purification and extraction of heavy metals.

## 2. Materials and Methods

### 2.1. Theoretical Methods

**Figure 1:**
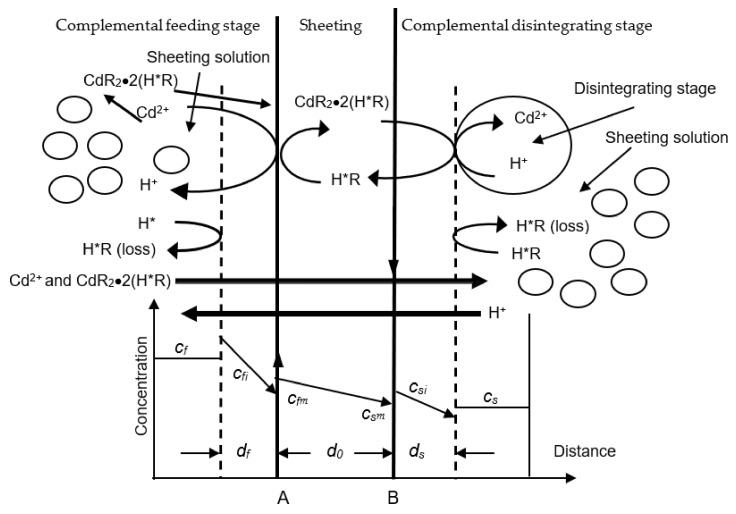
Principle schematic of Cd(II) dislodging through the diphasic sheeting device. H*R represents the Cyanex-301 within the sheeting. CdR_2_•2(H*R) represents the organic metallic compounds, H^+^ is the hydrogen ion, and Cd(II) represents the divalent cadmium ions (A and B are the sheeting borderlines).

Figure 1 is the theory of DSD, in which dislodging procedures and molarity varieties are depicted.

The DSD system consists of three stages: (1) the complemental feeding stage including metallic ions and sheeting liquor, (2) the sheeting stage that is implanted by PVDF (Kynoar, Polyvinylidene fluoride) sheet, impregnated with an organic liquor including Cyanex-301 and dissolvant, with sheeting serving as a uniform barrier between two stages, and (3) complemental disintegrating stage with sheeting liquor and disintegrating dissolvant in which the metallo-ion is disintegrated from sheeting stage [12,13].

In this paper we propose a mechanism for the dislodging of metallo-ions through the DSD. The following steps are necessary:

(1) The transferring of metallo-ions from the complemental feeding stage to the interfacial layer of feeding-sheeting stage.
(2) On the feeding side interfacial layer of the DSD, dislodging of the divalent metallo-ions from liquid liquors including Cyanex-301 may be represented as the Equation (1) below.

(1) $$M_{f}{}^{2+}+\frac{m+n}{2}{\left(H*R\right)}_{2,\mathrm{org}}\overset{K_{1}}{\underset{K_{-1}}{⇄}}MR_{n}.mH*R_{\left(\mathrm{org}\right)}+nH_{f}{}^{+}$$

where *f*, M_f_^2+^ and org represent complemental feeding stage, divalent metallo-ions, and organic stage, and (H*R)_2_ denotes the dimeric form of active carriers such as Cyanex-301.

(3) The metallic complex has been transferred through the sheeting A–B.
(4) At the other side of the interfacial layer of the sheeting, the metallic complex is dissolved sheeting liquor and the metallo-ions are resolved by disintegrating dissolvant. The chemical reaction can be expressed as the Equation (2) below.

(2) $${\mathrm{MR}}_{n}.mH*R_{\left(\mathrm{org}\right)}+nH_{s}{}^{+}\overset{K_{2}}{\underset{K_{-2}}{⇄}}M_{s}{}^{2+}+\frac{m+n}{2}{\left(H*R\right)}_{2,\mathrm{org}}$$

where s represents complemental disintegrating stage. K_1_, K_−1_, K_2_, K_−2_ are the pseudo-first-order rate constants of the forward and backward reactions of two interfacial layers.

(5) Carrier Cyanex-301 returns from B to A [11,12,13,14,15].

In this mechanism, the dislodging of Cd(II) across DSD is interpreted by considering only transferring parameters, because the complex reaction between the Cd(II) and Cyanex-301 in the interfacial layers is faster compared to the transferring in the liquid and sheeting stage [4,13,17]. Then, in this model, the dislodging of Cd(II) in the DSD procedure can be considered as four sequential steps. If the transferring procedure is interpreted by the Fick^‘^ theorem, the dislodging percentage of each step can be represented as follows:

(3) $$J_{f}=\frac{D_{f}}{d_{f}}(c_{f}-c_{fi})$$

*D_f_*, *d_f_*, *c_f_* and *c_fi_* represent transferring parameters and the liquid thickness and sheeting interfacial layer, the molarity of Cd(II) in complemental feeding stage, and the molarity of Cd(II) in liquid and sheeting interfacial layer.

(4) $$J_{i0}=K_{1}c_{fi}-K_{-1}\bar{c_{f}}$$

where $\bar{c_{f}}$ illustrates the molarity of Cd(II) in the sheeting stage.

(5) $$J_{0}=\frac{D_{0}}{d_{0}}(\bar{c_{f}}-\bar{c_{s}})$$

where $\bar{c_{s}}$, *D*_0_ and *d*_0_ represent the molarity of Cd(II), transferring parameters and thickness in sheeting stage.

(6) $$J_{os}=K_{2}\bar{c_{s}}\frac{V_{0}^{}}{V_{s}}$$

where vs. and *V*_0_ represent volume of disintegrating stage in complemental disintegrating stage and sheeting stage.

Then, in stable situation, above dislodging percentages are equal [7,12,19,20,21,22]. Then, by associating Equations (1)–(6),

(7) $$J=\frac{K_{d}.c_{f}}{K_{d}D_{f}{}^{-1}+D_{0}{}^{-1}+\frac{1}{K_{2}}\frac{V_{s}}{V_{0}}+{\left(K_{-1}\right)}^{-1}}$$

can be expressed as $K_{d}=K_{1}/K_{-1}$.

Equation (7) can also be expressed as:

(8) $$J=P_{c}.c_{f}$$

The *c_f_* represents the molarity of complemental feeding stage. *P_c_* represents the permeability constant, which is gotten as:

(9) $$P_{c}=\frac{K_{d}}{K_{d}\frac{d_{f}}{D_{f}}+\frac{d_{0}}{D_{0}}+\frac{1}{K_{2}}\frac{V_{s}}{V_{0}}}$$

Equation (10) is received from velocity constant and Equation (1).

(10) $$K_{1}=\frac{{\left[H^{+}\right]}^{n}[{\mathrm{MR}}_{n}.mH*R]}{[M^{2+}]{\left[{\left(H*R\right)}_{2}\right]}^{\frac{m+n}{2}}}=\frac{K_{d}.{\left[H^{+}\right]}^{n}}{{\left[{\left(H*R\right)}_{2}\right]}^{\frac{m+n}{2}}}$$

By associating Equation (10),

(11) $$\frac{1}{P_{c}}=\frac{d_{f}}{D_{f}}+(\frac{d_{0}}{D_{O}}+\frac{V_{s}}{K_{2}V_{0}})\frac{1}{K_{1}}\frac{{\left[H\right]}^{n}}{{\left[{\left(H*R\right)}_{2}\right]}^{\frac{m+m}{2}}}$$

The velocity of disintegrating is very fast in the DSD, so $1/K_{2}$ can be ignored [12].

So we can simplify Equation (11) as:

(12) $$\frac{1}{P_{c}}=\Delta_{f}+\Delta_{o}\frac{1}{K_{1}}\frac{{\left[H^{+}\right]}^{n}}{{\left[{\left(H*R\right)}_{2}\right]}^{\frac{m+n}{2}}}$$

We can presume $\Delta_{f}$ and $\Delta_{o}$ as:

(13) $$\Delta_{f}=\frac{d_{f}}{D_{f}},$$

(14) $$\Delta_{0}=\frac{d_{0}}{D_{0}}$$

The relevance between [H^+^]^n^ and 1/*P_C_* is tested to be linear at the same carrier molarity. Therefore, the transferring constant of Cd(II) in the sheeting and the thickness of transferring layer between the complemental feeding stage and sheeting stage can be gotten with the linear slope method. In the same way, the relevance between [(H*R)_2_]^−2^ and *1*/*P_C_* is tested to be linear at the same H^+^ molarity in the complemental feeding stage [12,13].

Based on the definition of permeability constant, dislodging flux of sheeting can also be expressed as:

(15) $$J=-\frac{V_{f}}{A}(\frac{dc_{f}}{d_{t}})=P_{c}.c_{f}$$

where *V_f_* represents the volume of complemental feeding stage and *A* represents impact area of sheeting.

Integrating Equation (15), the following formula can be gotten:

(16) $$\ln\frac{c_{f(t)}}{c_{f(0)}}=-\frac{A}{V_{f}}P_{c}.t$$

The c*_f_*_(0)_ and *c_f_*_(*t*)_ represent the molarity of Cd(II) in the complemental feeding stage at *t* = 0 and *t* = *t*. Equation (16) represents the *P_c_* value as the straight line with slope in the figure that is gotten in various running conditions.

To formulate the model, the following assumptions are also made:

(1) The Cd(II) transfers in the organic medium only as the CdR_2_•2(H*R) complex.
(2) There is no net flow on account of convection within the fluid sheeting.
(3) The metallo-ions react only with Cyanex-301 at the sheeting interfacial layers.
(4) The Cyanex-301 monomer and dimer are in equilibrium at all times throughout organic stage [12].
(5) The solubility of Cyanex-301 in liquid acidoc fluid is not considered, and therefore its molarity in DSD is assumed to remain unchanged [13,23,24,25,26].

Metallic complex (CdR_2_•2(H*R)) dislodging through the sheeting may be interpreted by Fick’s second theorem:

(17) $$\frac{\partial C_{\mathrm{CdR}}}{\partial t}=D_{\mathrm{CdR}}\frac{\partial^{2}C_{\mathrm{CdR}}}{\partial x^{2}}$$

where *C*_CdR_ represents molarity of the CdR_2_•2(H*R) complex in the organic liquor; *D*_CdR_ represents diffusivity of the CdR_2_•2(H*R) complex; *x* represents distance in the fluid sheeting. The borderline conditions in each of the DSD–liquid interfacial layers represent the equality of the free Cd(II) mass transferring to or from the interfacial layers in the liquid stage with the transferring of the organic–metallic Cd(II) complexes into or out of the organic stage in each borderline:

(18) $${\left.D_{\mathrm{CdR}}\frac{\partial C_{\mathrm{CdR}}}{\partial x}\right|}_{A}=k(C_{\mathrm{CdR}}^{0}-C_{\mathrm{CdR}}^{A})$$

(19) $${\left.D_{\mathrm{CdR}}\frac{\partial C_{\mathrm{CdR}}}{\partial x}\right|}_{A}=k(C_{\mathrm{CdR}}^{B}-C_{\mathrm{CdR}}^{0})$$

where *k* represents mass transferring constant in the liquid stage at borderline A or B; *C*_CdR_^0^ represents equivalent CdR_2_•2(H*R) molarity corresponding to the bulk liquid stage Ce(IV) molarity; *C*_CdR_^A^ represents interfacial CdR_2_•2(H*R) molarity at interfacial layer A; *C*_CeR_^B^ represents interfacial *C*dR_2_•2(H*R) molarity at interfacial layer B; A, B represents dislodging and disintegrating borderlines, respectively. Given that the CdR_2_•2(H*R) complex does not exist in the liquid stage, the mass transfer driving force of Cd(II), as Cd^2+^, must be estimated by converting the liquid stage molarities to corresponding organic stage molarities *C*_CdR_^0^, using the dislodging equilibrium constant. Therefore, it is possible to calculate the equivalent CdR_2_•2(H*R) molarity for the most liquid-free Cd^2+^ in the following:

From Equation (10), we get:

(20) $$C_{\mathrm{Cdr}}^{0}={\left[{\mathrm{CdR}}_{2}•2(H*R)\right]}_{\mathrm{most}}=K_{1}\frac{{{[\mathrm{Cd}}^{2+}]}_{\mathrm{most}}{{\left[(H*R\right)}_{2}]}^{3}}{{{[H}^{+}]}^{2}}$$

where [Cd^2+^]_most_ represents uncomplexed Cd(II) in the most liquid liquor; [CdR_2_•2(H*R)]_most_ represents CdR_2_•2(H*R) molarity of organic stage in equilibrium with the most liquid stage; [(H*R)_2_] represents Cyanex-301 dimer molarity in the organic stage. Besides, for describing Cyanex-301 transferring through the DSD, the different compounds that include the extractant are in equilibrium and must be grouped together. Since the Cyanex-301 dimer and the Cd(II) complex include 6 and 2 Cyanex-301 molecules, respectively, the total Cyanex-301 transport percentage may be expressed as:

(21) $$\frac{\partial C_{\left(H*R,\mathrm{DR},\mathrm{Cd}R\right)}}{\partial t}=D_{H*R}\frac{\partial^{2}C_{H*R}}{\partial x^{2}}+2D_{\mathrm{DR}}\frac{\partial^{2}C_{\mathrm{DR}}}{\partial x^{2}}+6D_{\mathrm{CdR}}\frac{\partial^{2}C_{\mathrm{CdR}}}{\partial x^{2}}$$

where *C*_(H*R, DR, CdR)_ represents Cyanex-301 total = *C*_H*R_ + *C*_DR_ + 6*C*_CdR_; *C*_H*R_ represents molarity of the Cyanex-301 monomer; *C*_DR_ represents molarity of the Cyanex-301 dimer. Since no Cyanex-301 enters the liquid stages, in any form, its transferring at both interfacial layers is considered to be negligible and the borderline conditions at A and B may be expressed as follows:

(22) $${\left.D_{H*R}\frac{\partial C_{H*R}}{\partial x}+2D_{\mathrm{DR}}\frac{\partial C_{\mathrm{DR}}}{\partial x}+6D_{\mathrm{PbR}}\frac{\partial C_{\mathrm{PbR}}}{\partial x}\right|}_{A,B}=0$$

The initial conditions represent the uniformity of the Cyanex-301 monomer and dimmer molarities, as well as the absence of Cd(II), throughout the DSD:

Then for all *x*,

(23) $$C_{\mathrm{CdR}}=0$$

(24) $$C_{H*R,\mathrm{DR}}={\left[\mathrm{Cyanex}-301\right]}^{0}$$

where [Cyanex-301]^0^ represents initial molarity of Cyanex-301.

To address this model, the liquid–liquid mass balance and species distribution must be determined before proceeding with transferring the liquid for formulation. Since the dislodging and disintegrating liquors include impressive amounts of sulfate, hydroxide, and hydrogen ions, much of the Cd(II) presents as complexes with above ions. The free (uncomplexed divalent ions) Cd(II) molarity must be calculated in order to evaluate the borderline conditions with Equation (20), each time [13]. Following this, the transferring formulas for Cd(II) and total Cyanex-301 are discretized applying the limited differential skill as proposed from Hoffman [12,25]. The nonlinear algebraic formulation set formed by the transfer formulation and boundary conditions is solved by the method of Newton Raphson. Finally, Cd(II) dislodging from the dislodging stage and added to the disintegrating stage is calculated using differential mass balances [12,13,14].

(25) $${\left.D_{\mathrm{CdR}}\frac{\partial C_{\mathrm{CdR}}}{\partial x}\right|}_{A,B}=\frac{V}{A}\frac{dC_{\mathrm{Cd}}}{dt}$$

The *V* represents volume of the liquid stage; *A* represents area of the liquid–organic interfacial layer; *C*_Cd_ represents total Cd(II) molarity in the liquid stage.

Hydrogen ions from unreacted cyanex-301 flow in opposite directions and balance the charge of Cd(II) lost or obtained in each liquid stage.

(26) $${\left.\frac{dC_{H}}{dt}\right|}_{A,B}=-2{\left.\frac{dC_{\mathrm{Cd}}}{dt}\right|}_{A,B}$$

where *C*_H_ represents total hydrogen ion transferred into or out of the liquid stage. Then, the whole process is repeated [12,13].

The model presented may easily be applied for a continuous procedure by eliminating the time dependency of Equations (17) and (21), and converting the liquid stage mass balances, Equation (25), to a flow scenario.

(27) $${\left.D_{\mathrm{CdR}}\frac{\partial C_{\mathrm{CdR}}}{\partial x}\right|}_{A,B}=\frac{V_{A}}{A_{A}}\frac{dC_{\mathrm{Cd}}}{dz}$$

where *V*_A_ represents volumetric flow of liquid liquor, A or B; *A*_A_ represents area of the liquid–organic interfacial layer per distance of tangential flow; *z* represents distance in the direction of flow. Despite these varieties, the dislodging velocity will still be controlled by the dislodging through the DSD and therefore subject to the same restrictions as found in the nonstationary state case [11,12,13,14].

### 2.2. Reagents and Instruments

The cadmium nitrate (Cd(NO_3_)_2_) from the simulated industrial effluent according to the actual effluent of metal ore in a province, HAc/NaAc (buffer solution) and PAR [4(2-pyridyla20) resoroin] used in previous work, is set to analytical grade. All chemical reagents are dissolved by deionized water. The Bis (2,4,4 trimethylamyl) dithiophosphonic acid (Cyanex-301) is a commercial extractant (purity > 96%) and used without any further purification. Petroleum is washed with concentrated sulfuric acid and distilled at 190–210 °C.

**Figure 2:**
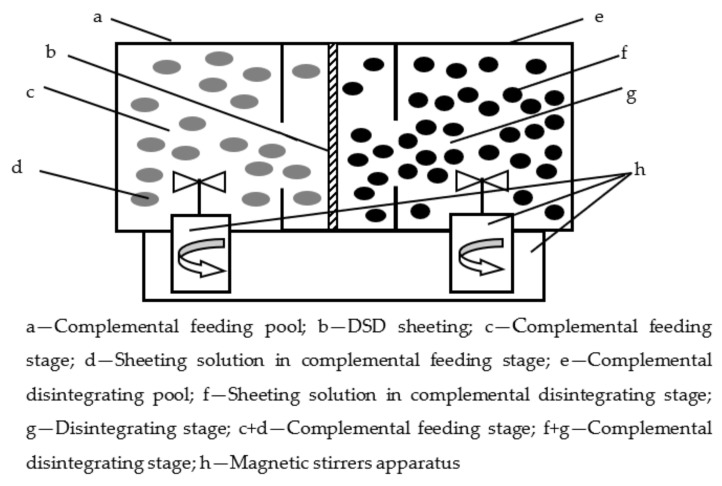
Experimental installation of DSD procedure.

The experimental device of this study is shown in Figure 2 as below. The experimental device is divided into two cells, each with 110 mL. Two cells are partitioned by the PVDF (Kynoar, Polyvinylidene fluoride) sheet, which has a 69% porosity, 66 μm thickness, 0.46 μm pore size, and 1.81 curvature. The impactive area is 12.0 cm^2^. The JJ-1 accurate strengthening electro-stirrers are from Shaanxi, China. The UV-1200 spectrophotameter is from Shanghai, China [12,13].

### 2.3. Experimental Procedure

The complemental feeding stage is a liquid liquor that includes Cd(II) and sheeting liquor. The metal liquor is prepared by dissolving the required amount of Cd(NO_3_)_2_.4H_2_O. The complemental disintegrating stage is the mixture of liquid liquor including hydrochloric acid liquor and sheeting liquor. The sheeting liquor is prepared by dissolving of Cyanex-301 in petroleum. The PVDF (Kynoar) film as support is pre-wetted with the required amount of sheeting liquor for more than 6 h in order to make the pores fill with Cyanex-301. The experiments are performed in the pH scope of 3.0–5.6 to investigate the impact of pH of complemental feeding stage on dislodging, the hydrogen ion molarity of complemental disintegrating stage, the volumetric ratio of sheeting liquor and hydrochloric acid liquor and molarity of Cd(II) of complemental feeding stage. The pH of complemental feeding stage at each section of experiment is kept constant using buffer liquors during other conditional experiments [12,13].

The Cd(II) molarity is analyzed by spectrophotography (580–590 nm) using developer-PAR. A precision digital ion-meter (Phs-3c) with a compound glass electrode is applied to pH value measuring (+0.01 pH) [12,13].

## 3. Results and Discussion

### 3.1. Impact of Temperature

**Figure 3:**
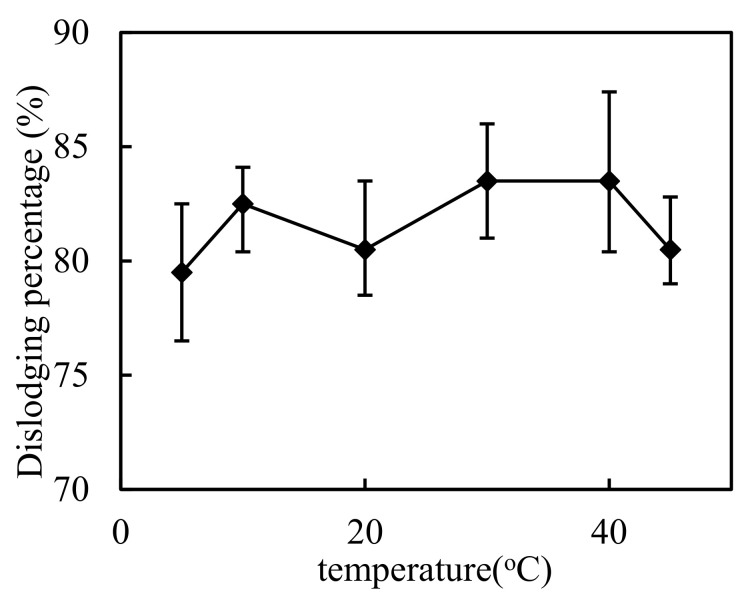
The impact of temperature.

First, we investigated the impact of room temperature on the DSD system. The assumed conditions of the test were chosen at a specific pH value of the complemental feeding stage, which was configured to 4.50. The molarity of Cd(II) was set to 2.60 × 10^−4^ mol/L, the volumetric ratio of sheeting liquor and complemental feeding liquor(S/F) was 1:2 in the complemental feeding stage, the volumetric ratio of sheeting liquor and hydrochloric acid liquor (S/D) was 2:1, the hydrochloric acid molarity was 3.50 mol/L, and the Cyanex-301 molarity was 0.100 mol/L in the complemental disintegrating stage. We adjusted the temperature chamber to 5 °C, 10 °C, 20 °C, 30 °C, 40 °C and 45 °C. The results are represented in Figure 3 below. When the temperature reached 45 °C, the dislodging percentage decreased slightly, because if the temperature is too high, the organic solution on the sheet surface falls off. It was obvious that the color of the membrane had changed, so this conclusion is valid. In the procedure of practical application, the room temperature cannot reach more than 40 °C. Therefore, we can conclude that the dislodging percentage of the system is not significantly affected by temperature at ordinary room temperature.

### 3.2. Constancy of DSD

To define constancy of DSD compared with a traditional sheeting device, the tendency of Cd(II) molarity varieties in the complemental feeding stage and complemental disintegrating stage with time were investigated under defined running conditions, long time.

**Figure 4:**
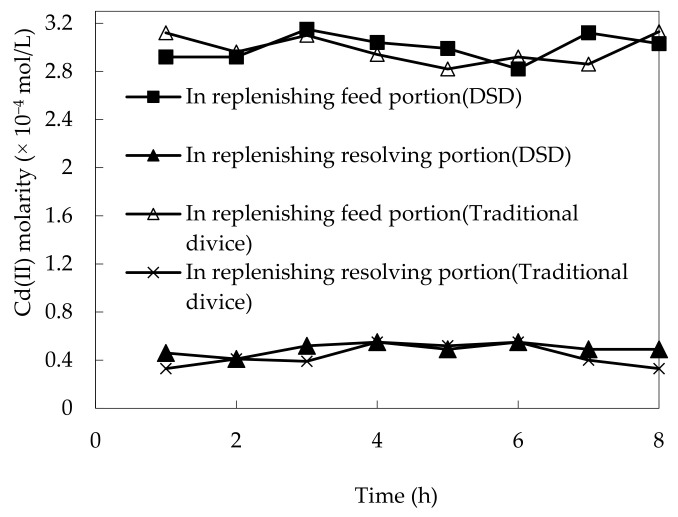
The constancy of DSD.

The assumed conditions of test were chosen at a specific pH in the complemental feeding stage, which was configured to 4.80. Initial molarity of Cd(II) was 2.60 × 10^−4^ mol/L, volumetric ratio of sheeting liquor and complemental feeding liquor(S/F) was 1:2 in the complemental feeding stage, the volumetric ratio of sheeting liquor and hydrochloric acid liquor(S/D) was 2:1, the hydrochloric acid molarity was 3.50 mol/L, and the Cyanex-301 molarity was 0.100 mol/L in the complemental disintegrating stage. The results are represented in Figure 4. After 60 min, the tendency of Cd(II) molarity varieties was constant, so we took a sample over 60 min in each dislodging experiment. We found that after 6.0 h, the Cd(II) molarity and stability of the disintegrating stage decreased gradually under use of the traditional sheeting device and the Cd(II) molarity in both feeding stage and complemental disintegrating stage remained constant under usage of DSD. This is because the carrier in traditional sheeting devices diminishes gradually and DSD in the complemental stage can complement Cyanex-301 in sheeting [23,24]. This part of the procedure was to study and compare the stability of new technology and old technology. After several experiments, we found that the new technology could maintain stability for a longer time. Because there is a supplement solution, it can supplement the organic solution dropped from the membrane body due to long-time operation. We can conclude that DSD is more constant than the traditional sheeting device. However, if the operation continues, the organic solution Cyanex-301 on the sheet will still fall off, because the sheet itself has a life. Therefore, one sheet will only be used about 15 times, so as to ensure the accuracy of data and the effectiveness of future applications.

### 3.3. Impact of the Volumetric Ratio of Sheeting Liquor and Feeding Liquor(S/F)

The volume of sheeting liquor and feeding liquor has an equilibrium value on the dislodging percentage. The sum of sheeting liquor and feeding liquor is certain: more sheeting liquor, less feeding liquor. Similarly, more feeding liquor means less sheeting liquor. More sheeting liquor and less feeding liquor lead to more carriers and fewer analyzers, which will slow down the analytical reaction, slow down the overall process and reduce the removal rate. Similarly, with less sheeting liquor and more feeding liquor, fewer carriers and more analyzers, the complexation reaction will slow down, the overall process will also slow down, and the dislodging percentage will still decrease. Therefore, the best volumetric ratio of sheeting liquor and feeding liquor is an important condition to determine the highest dislodging percentage [13,14].

**Figure 5:**
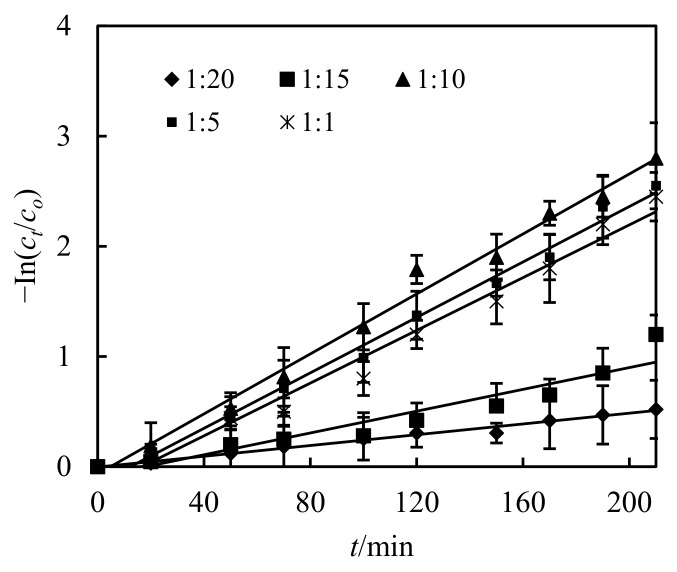
Impact of volumetric ratio of sheeting liquor and feeding liquor on dislodging of Cd(II).

The impact of volumetric ratio of sheeting liquor and feeding liquor(S/F) was investigated in this section. All other parameters such as pH, molarity of Cd(II) in the complemental feeding stage, molarity of hydrochloric acid liquor, and the volumetric ratio of sheeting liquor and hydrochloric acid liquor(S/D) in the complemental disintegrating stage were kept constant at 4.8, 2.6 × 10^−4^ mol/L, 4 mol/L and 2. The impact of the volumetric ratio of sheeting liquor and feeding liquor(S/F) in the complemental feeding stage on dislodging of Cd(II) is represented in Figure 5. Law of curve in the figure illustrates that the Cd(II) dislodging percentage decreased with the increasing of the volumetric ratio from S/F 0 to 1.0. When the volumetric ratio of S/F was 0.1, the Cd(II) dislodging percentage was 85.3%. Considering saving chemical reagents as well as increasing dislodging percentage, we chose 0.1 mol/L as the best volumetric ratio for the following tests [12].

### 3.4. Impact of pH in the Complemental Feeding Stage

**Figure 6:**
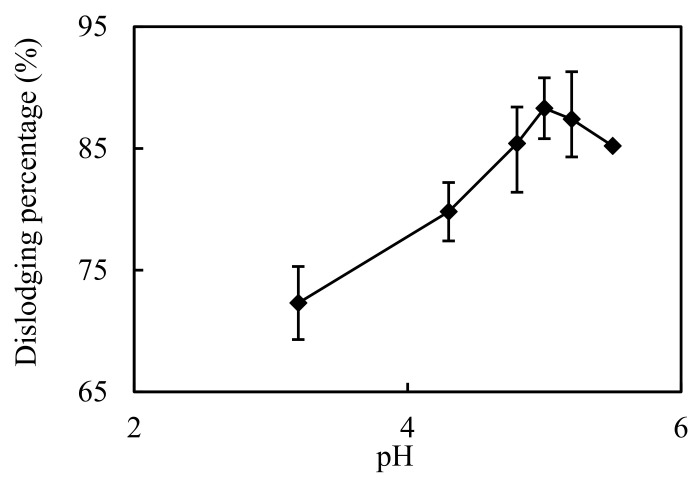
Impact of pH in the complemental feeding stage on dislodging of Cd(II).

The impact of pH on Cd(II) permeating and dislodging was investigated in the pH scope 3.2~5.5, that was configured with an HAc/NaAc buffer liquor. Volumetric ratio S/F was configured to 0.1. Cd(II); molarity in the complemental feeding stage was 2.6 × 10^−4^ mol/L. The molarity of hydrochloric acid in complemental disintegrating stage was 2.0 mol/L and the S/D was 3. The results are represented in Figure 6. The Cd(II) percentage increased as the pH of the feeding liquor increased from 3.0 to 5.0, and the maximum Cd(II) dislodging percentage observed at a pH of 5.0 was 88.7%. Above pH 5.0 of feeding liquor, the Cd(II) percentage decreased. This is similar to the pH influence on the distributive constant of the dislodging procedure [12,23]. This is mainly because the dislodging procedure is mainly dominated by the mass-transferring driving force caused by the distributive equilibrium, when the complemental impact of the fluid sheeting and the transferring Cd(II) ions’ mobility are ascertained under specific test conditions [12].

### 3.5. Impact of Initial Molarity of Cd(II) in the Complemental Disintegrating Stage

**Figure 7:**
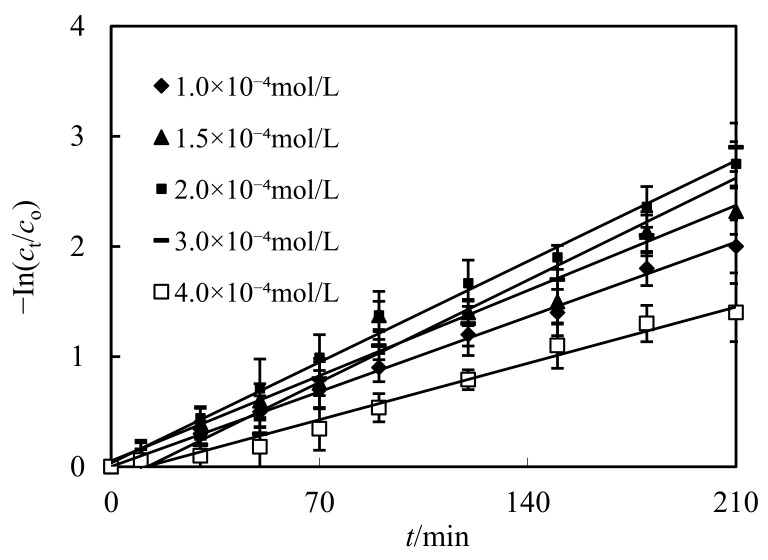
Impact of initial molarities of Cd(II) on dislodging of Cd(II).

The impact of Cd(II) molarity on percentage and dislodging factor Cd(II) was investigated within the scope of 1.0 × 10^−4^~4.0 × 10^−4^ mol/L. The PH of the complemental feeding stage was configured to 5.0. Volumetric ratio S/F was configured to 0.10. Volumetric ratio S/D was configured to 3 and hydrochloric acid was configured to 4.0 mol/L in the complemental disintegrating stage. The results gotten are presented in Figure 7. With increasing of Cd(II) molarity in the complemental feeding stage from 1.0 × 10^−4^ mol/L to 3.0 × 10^−4^ mol/L, the Cd(II) percentage increased, then decreased, while the initial molarity of Cd(II) increased in the complemental feeding stage. Within this molarity scope of Cd(II) in the complemental feeding stage, the availability of Cd(II) at the feeding–sheeting interfacial layer increased with increasing Cd(II) molarity. However, when the Cd(II) molarity in the feeding liquor become higher in comparison to Cyanex-301 molarity in the sheeting stage, the Cd(II) percentage decreased. This illustrates that the amount of moles transported through the sheeting per unit area of the sheeting per unit time are ascertained when the molarity of Cyanex-301, the impact area of sheeting, and time are ascertained [12,13].

### 3.6. Impact of the Volumetric Ratio of Sheeting Liquor and Hydrochloric Acid Liquor(S/D)

**Figure 8:**
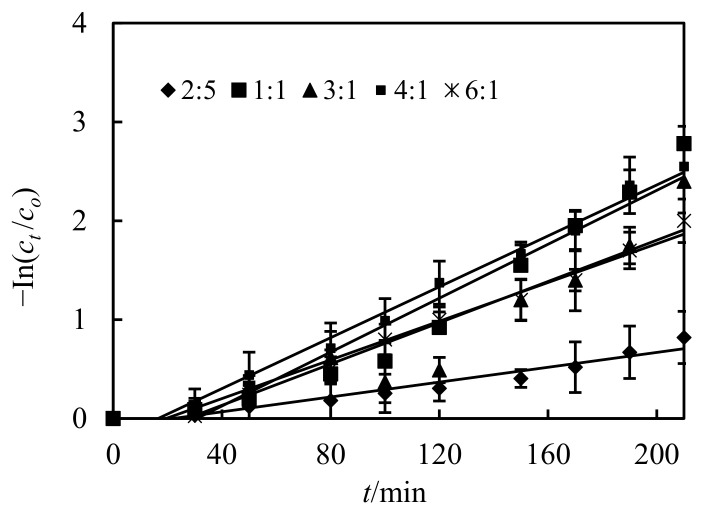
Impact of volumetric ratio of sheeting liquor and hydrochloric acid liquor on dislodging of Cd(II).

The impact of volumetric ratio of sheeting liquor and hydrochloric acid liquor(S/D) in the complemental disintegrating stage on Cd(II) dislodging percentage is represented in Figure 8. It illustrates that the Cd(II) dislodging percentage increased with an increasing of the volumetric ratio. When volumetric ratio of S/D increased, the droplets of the disintegrating liquor dispersed in the sheeting stage increased distinctly [4]. In this way, the sheeting stage and complemental disintegrating stage provide an extra disintegrating surface and promote renewal velocity of fluid sheeting, which leads to an extreme disintegrating velocity for the complemental feeding stage from the sheeting stage and extension of fluid sheeting life [12,13], which enhances the Cd(II) dislodging percentage.

Here we needed to pay attention to the selection of best conditions. We considered the price of materials and if removal efficiency was similar under the two conditions. We chose the condition that used less organic sheeting liquor, because sheeting liquor is very expensive, and at the same time much sheeting liquor is not stable. Similarly, if the dislodging efficiency was similar under different acidity or alkalinity conditions, we chose the condition with less acid or alkali, because many acids and bases are unstable.

### 3.7. Impact of the Molarity of Hydrochloric Acid in the Complemental Disintegrating Stage

The disintegrating reaction at the sheeting-disintegrating stage plays a vital role in the transfer of metallo-ions from the complemental feeding stage to the complemental disintegrating stage. Therefore, the impact of molarity of hydrochloric acid was investigated in the investigation.

The greater the acidity of the complemental disintegrating stage, the greater the molarity difference between the two sides, and the greater the migration power of metal ions. However, if the acidity is too high, the organic solution on the sheeting easily falls off. Therefore, this study looked to get the best acidity [14].

**Figure 9:**
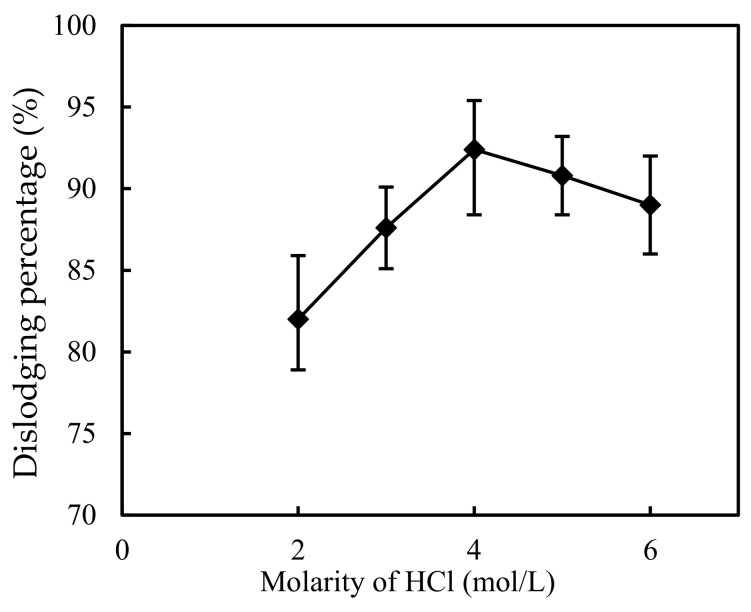
Impact of molarities of hydrochloric acid in the complemental disintegrating stage on dislodging of Cd(II).

The parameters, such as pH, molarity of Cd(II), S/F of the complemental feeding stage, and the S/D, were configured to 0.100, 5.0, 2.6 × 10^−4^ mol/L and 1. Figure 9 shows the impact of the dislodging velocity of Cd(II) in different molarities of hydrochloric acid. It illustrates that the Cd(II) dislodging percentage increased with the increasing of acid molarity. By further increasing hydrochloric acid molarity from 4.0 mol/L to 6.0 mol/L, the Cd(II) dislodging percentage decreased because the amount of complex of Cd(II) and Cyanex-301 which dislodge through the sheeting per unit area of the sheeting per unit time are ascertained when the Cyanex-301 molarity, the Cd(II) molarity of the complemental feeding stage, and the impact area of sheeting and time are ascertained [13,14,24]. Therefore, the molarity of hydrochloric acid in the complemental disintegrating stage was 4.0 mol/L as the optimal condition.

### 3.8. Impact of Ion Intensities in the Complemental Feeding Stage

**Figure 10:**
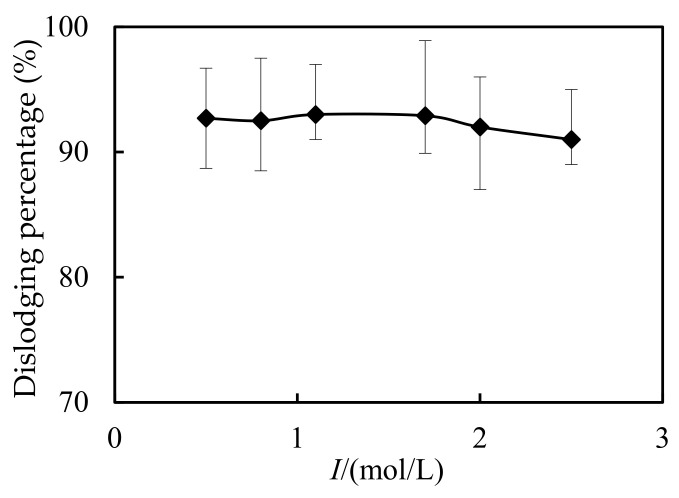
Impact of ion intensities on dislodging of Cd(II).

In industrial slops, there always exist other ions that may affect the dislodging of Cd(II). Therefore, the impact of ion intensities in the complemental feeding stage on the Cd(II) dislodging percentage was investigated. Thus NaCl and KNO_3_ were used to simulate the industrial slops, and investigate the impacts of the initial ion-density in the feeding cell on the dislodging percentage of Cd(II). As shown in Figure 10, the dislodging percentage of Cd(II) increased when the initial ion-density changed from 0.4 mol/L to 1.5 mol/L, and kept higher than 90%. This result shows that during the removal process other ions in the feeding cell have little effect on the efficiency of the technical system, which is consistent with previous research results [14,33].

### 3.9. Retention in Sheeting Stage

**Figure 11:**
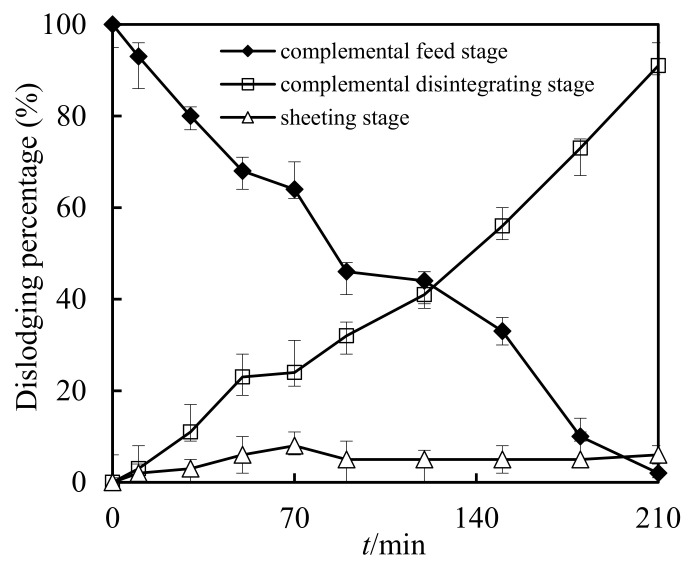
Retention in sheeting stage and impact of disintegration.

In previous studies using SLM to dislodge heavy metals, the retention of heavy metal ions on the sheeting was observed and concerning [14,37]. In this work, Cd(II) ions also received attention. Their retention in the sheeting stage and the impact of disintegration was investigated in the best running requirements. The volumetric ratio S/F was configured to 0.10, initial molarity of Cd(II) was 2.60 × 10^−4^ mol/L in the complemental feeding stage, volumetric ratio S/D was configured to 2.00, pH value was configured to 5.00, and molarity of hydrochloric acid liquor was also configured to 5.00 mol/L in the complemental disintegrating stage. The molarity of Cd(II) on the sheeting can be calculated according to the molarity of Cd(II) in the feed cell and the disintegrating cell. As shown in Figure 11, the adsorption of Cd(II) on the sheeting increased with running time. However, with the prolongation of the operation time, the increase rate decreased, which led to a stable Cd(II) percentage on the sheeting when the operation time exceeded 210 min.

## 4. Dynamic Analysis

**Figure 12:**
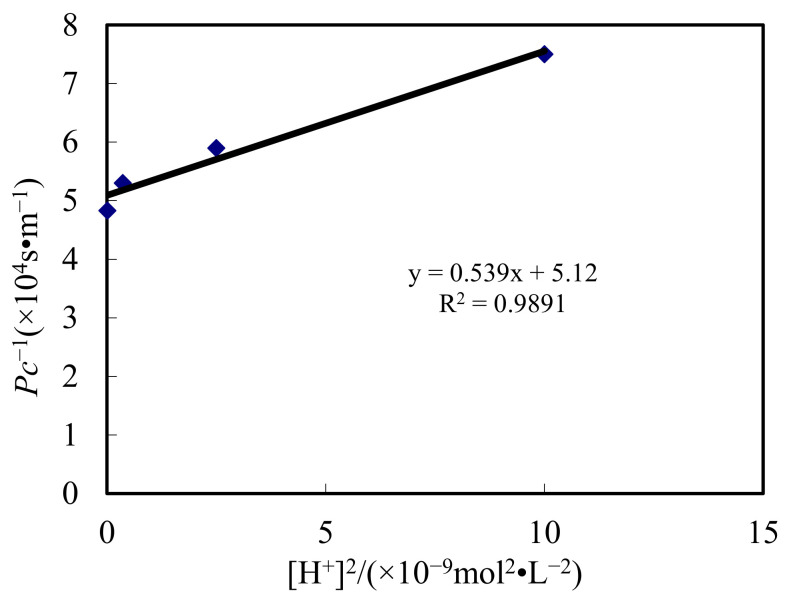
Fitting of test results and theoretical derivation.

With the data on the pH impact of the complemental feeding stage, the relevance of *1*/*P_C_* and [H^+^]^2^ was explored. When Cyanex-301 molarity is ascertained, *P_C_* is ascertained. The law of results is represented in Figure 12.

It illustrates the relevance of *1*/*P_C_* and [H^+^]^2^ is a linear relationship in a specific pH value. The R^2^ is 0.98, which is identical to the theory with Equation (12). The gradient and nodal increment are 5.39 × 10^12^ and 5.12 × 10^4^. Transferring layer thickness *d_f_*, calculated by the transferring constant of Cd(II) in the liquid that is 6.92 × 10^−10^ m^2^/s [38], is 1.08 × 10^−4^ m. The *K_1_*, which can be ascertained by dislodging experiment, is 2.7 × 10^−8^. Then, the *D_o_* of the transferring constant in the sheeting, gotten by combined Equations (13) and (14), is 1.31 × 10^−7^ m^2^/s.

The new dynamic formula is ascertained by *d_f_* and *D_o_* in the DSD combination. It can be represented as

(28) $$P_{C}=\frac{1}{5.12\times 10^{4}+5.39\times 10^{12}{\left[H^{+}\right]}^{2}}$$

## 5. Conclusions

The dislodging of Cd(II) in industrial effluent with DSD using Cyanex-301 as mobile Cyanex-301 was investigated. The following conclusions were drawn from the above study.

(1) The optimal conditions for dislodging Cd(II) were that the molarity of hydrochloric acid was 4.0 mol/L, S/F was 1:10, Cyanex-301 molarity was 0.150 mol/L, and S/D was 1:1 in the complemental disintegrating stage, initial molarity of Cd(II) was 3.2 × 10^−4^ mol/L, and optimal pH was 5.0 in the complemental feeding stage; when dislodging time was 210 min, the dislodging percentage was 92.9%.
(2) Through this study, a relevance (modal) was established to elaborate the metallo-ion reaction and dislodging in the DSD. A new dynamic formula was inferred. The transferring constant of the sheeting stage and transferring layer thickness of the complemental feeding stage were gotten by using Straight line and Gradient method. They were 1.31 × 10^−7^ m^2^/s and 1.08 × 10^−4^ m.
(3) In the DSD, large amounts of sheeting liquor were used; this could be complemented by the loss of Cyanex-301 in the sheeting device. As a result, the Cd(II) dislodging percentage is increased, the constancy of sheeting is increased, and the life of the sheeting is extended.

At present, research is still in the experimental stage in the laboratory. After experimental research, this technology may be applied in practice. The selected optimal operation conditions could break through the bottleneck of water treatment and sheeting technology. Future research needs to focus on the various materials of the device, reducing costs, and optimizing the process for pilot study.

We could apply the extracted cadmium to the alloy industry, the electroplating industry, and battery manufacturing. It could make great contributions to the country’s industrial development. Of course, this requires the joint efforts of our various industries, good national policies, cost reduction, and the establishment of a good bridge for cadmium recycling. It could also provide scientific and theoretical support for the treatment of industrial waste residue, especially the purification and extraction of heavy metals.

## Data Availability

This study does not report any data.
